# The Brainard Medical Society

**Published:** 1868-05-01

**Authors:** I. B. Washburn

**Affiliations:** Secretary


### THE BRAINARD MEDICAL SOCIETY.

The society met in the Court House in Winamac, Indiana, April 6, 1868. The president, Dr. F. B. Thomas, in the chair. Minutes read and adopted.

The annual election of officers resulted as follows:

*President*—F. B. Thomas; *Secretary*—I. B. Washburn; *Treasurer*—A. M. Pearson; *Censors*—Drs. Wm. Kelsey, Wm. T. Cleland, and J. W. C. Eaton.

Dr. R. W. Jackson, of Monterey, Indiana, was admitted to membership.

Drs. L. D. Glazebrook, W. T. Cleland, I. B. Washburn, and J. B. Hoag were elected delegates to the State Medical Society.

Dr. Hoag read a paper on "Typhoid Fever."

Cases of convulsions were reported by Drs. Hoag, Thomas, and Cleland, and discussed by most of the members present.

Dr. Eaton was appointed essayist.

Apjourned to meet in Winamac, Indiana, Tuesday, May 5th, 1868.

I. B. WASHBURN, M.D., *Secretary.*

